# Application of Butterfly Clos-Network in Network-on-Chip

**DOI:** 10.1155/2014/102651

**Published:** 2014-01-29

**Authors:** Hui Liu, Linquan Xie, Jiansheng Liu, Lei Ding

**Affiliations:** ^1^Jiangxi University of Science and Technology, Ganzhou 341000, China; ^2^School of Information Science and Engineering, Jishou University, Jishou 416000, China

## Abstract

This paper studied the topology of NoC (Network-on-Chip). By combining the characteristics of the Clos network and butterfly network, a new topology named BFC (Butterfly Clos-network) network was proposed. This topology integrates several modules, which belongs to the same layer but different dimensions, into a new module. In the BFC network, a bidirectional link is used to complete information exchange, instead of information exchange between different layers in the original network. During the routing period, other nondestination nodes can be used as middle stages to transfer data packets to complete the routing mission. Therefore, this topology has the characteristic of multistage. Simulation analyses show that BFC inherits the rich path diversity of Clos network, and it has a better performance than butterfly network in throughput and delay in a quite congested traffic pattern.

## 1. Introduction

As an improvement on SoC (System-on-Chip), NoC is an intercommunication-based network system, which is implemented on an integrated circuit. With the development of IC technology, SoC based on the traditional bus architecture has been unable to meet the increasing requirement of network communication. With the transplantation of network technology from computer systems and the replacement of traditional bus structure with network structure, NoC solves the communication bottleneck issue of SoC and is rather promising. The topology of NoC defines the layout and on-link mode of nodes and lines in the network. As a key technology of NoC, it plays an important role in the performance of the network, such as throughput, delay, fault tolerance, and load balance. At present, total 100 number of research institutions and companies engaged in on-chip network research in which the better-known institutions have Satanford University in [1], Princeton University in [2], the University of Bologna, KTH [3], the SGS-Thomson semiconductor company, Arteriscompany France Curie University [4], and The Royal Swedish Institute of Technology, the Netherlands PHILIP laboratories.

NoC applications can be of a static or dynamic nature. In static applications, the parameters are well defined prior to the design phase, allowing a topology to meet the specific requirements needed by the given application during synthesis. In dynamic workload applications such as those dealt with in MPSoC scenarios, new application PEs may be inserted at any time. Regular topologies such as mesh and torus are often used to overcome such plug-and-play compatibility issues. Regular topologies however result in poor performance due to increases in power usage and hardware area overhead on account of the topological regularity. It has become evident through research that the application specific NoC architecture is superior to regular topologies in terms of power consumption and NoC resources [5]. Regular topologies tend to assume that NoC systems contain homogeneous cores, where in reality, many high performance SoCs make use of heterogeneous processors/cores. As a result, designs containing nonuniform core sizes do not match the standard, tile-based floor plan of these topologies [6]. For the majority of SoCs, it is known that sizes ranging from small to state-of-the-art systems can be designed with static (or semistatic) mapping of tasks to cores, and therefore the communication traffic characteristics of the SoC can also be obtained statically [7]. Given this fact, it is then possible to create a custom design that can cater to the communication characteristics of the system while reducing power and on-chip area and improving performance. This work addresses the static workload scenario, incorporating a number of factors during topology synthesis to generate application specific NoCs which meet the power and performance requirements of the given application.

With the increase of chip integration, the future number of transistors on a single chip will reach billions. On-chip multiprocessor is able to effectively use the massive on-chip transistors resources, which has become the development trend of high-performance processors [8–10]. Chip network has a more important influence in the scalability and performance of chip multiprocessor, which gradually becomes a research hotspot [11–13]. In recent years, on-chip network research has focused on low power design [14, 15], router design [16, 17], implementation and test methods [18, 19], and fault tolerance mechanism. Currently, few studies are about the topology and routing algorithm, and most of the existing single-chip multiprocessor uses the classical topology (including Mesh, Torus). As the size of chip network is usually smaller in scale than the large-scale parallel machines, on-chip network design must give full consideration to the difficulty of the physical realization, so the traditional network for parallel machines Mesh, Torus, and so forth does not necessarily apply to the classical topology. Thus according to its characteristics, studying for on-chip network topology is of great significance.

The remainder of this paper is organized as follows.  Section 2 provides some introductions on network topology architectures.  Section 3 presents the details about the routing algorithm design based on BFC architecture.  Section 4 presents the evaluation methodology and simulation results.  Section 5 summarizes the paper by presenting our conclusions in this area.

## 2. Topology Introductions

### 2.1. Clos Network Topology

**Figure 1:**
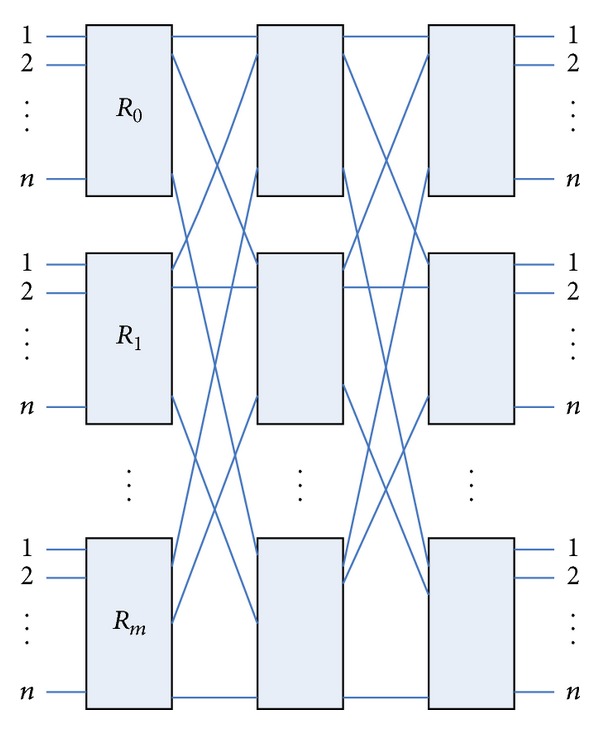
Three-Clos network topology.

Clos network [20–22] was first proposed by Charles Clos in 1953. Clos network is the most commonly used three Clos network *C*(*m*, *n*, *r*) shown in Figure 1, where *m* is the number of middle-class switching units; *n* is the input ports of the input stage switch module, which is the output ports of output level switching units; *r* is the number of input stage switching unit, which is the number of output stage switching units. Each switch module is a crossbar structure in accordance with crossbar model to work.

The cache architecture based on three Clos networks can be divided into three categories: full-cache-type Clos network, non-cache-type Clos network, and part-cache-type Clos network.

#### 2.1.1. Full-Cache-Type Clos Network (MMM, Memory-Memory-Memory)

The structure is based on three kinds of shared cache switching fabric. This uses a connection-oriented routing method. When the data packet arrives at the input stage switch modules, switching network based on its source input port and output port for the target opens up a transmission path routing. Because it is connection-oriented routing method, so this kind of transmission is a closed, nonblocking transmission. There are some caches in three-level MMM mode, and the routing can be used to record relevant information. However, this method requires a lot of middle-class switching module, the requirements of the size of the buffer space are very high, the bandwidth allocation and the buffer queue options are needed to solve the problem, and the operation is very complex. Therefore, the application of MMM is currently very narrow, and little has been put into actual business.

#### 2.1.2. Non-Cache-Type Clos Network (SSS, Space-Space-Space)

This kind of structure is a cache of the exchange without any structure. The exchange network design is relatively simple, which does not arrange the cache device. However, because it does not exist cache, the request for a new arrival, such as you need to complete before sending the output port and the use of intermediate-level switching unit selection, requires a more complex scheduling algorithms. Moreover, without caching devices, it is prone to internal blocking.

#### 2.1.3. Part-Cache-Type Clos Network (MSM, Memory-Space-Memory)

This kind of network uses the first- and third-level shared cache, and the exchange unit of the middle class does not use the cached exchange. It uses a packet-oriented routing. In the routing process, the routing operations are completed the input port buffer queue data packets in a cycle, and the selected packets are sent to the cache. In the next cycle it will be sent out, while other groups are rerouting operations experience.

Clos network has a good path diversity, but it may still occur in the internal block. Internal blocking is an effective request for the network, which is not a free intermediate module linked directly, and the so-called effective request is an empty request for input ports and output ports.

There are currently three different methods to ensure nonblocking network work, and the three methods are described as follows.

1. Strict sense nonblocking: for a *C*(*m*, *n*, *r*) network, when *m* ≥ 2*n* − 1 is established, which is called as strict nonblocking network. In a strict nonblocking state of the Internet, for any free port input stage and output stage, there is always a free link linked, which does not adjust to other multilevel network to establish a good path. Regardless of selection strategy will not appear blocked.
2. Wide sense nonblocking: the requirements of the wide sense nonblocking network are relatively low, and it refers to blocking within the network by routing algorithms to help solve the congestion problem and find the right link for the data packet without the need breaking the link for completed routing request.
3. Rearranged nonblocking: rearranged nonblocking network is an improvement for the generalized nonblocking network. It can be rearranged by using a generalized nonblocking mechanism to extend the network implementation, once the blockage occurs in the network by using a specific routing algorithm to break up the current network links based on the routing requests to reestablish the connection. This rearrangement algorithm can make the link to connect to rationalize and improve module utilization greatly. However, the design of corresponding algorithm is more complex which may re-break the link and make the cache delay, energy consumption largely. For a *C*(*m*, *n*, *r*) network, only when *m* ≥ *n*, the network is rearranged.In the actual network, the different types of networks have different roles. Our broad network of nonblocking Clos network routing algorithm provides a more efficient routing algorithm to improve the network performance. Nonblocking network can be rearranged, which can be used to study the reorganization algorithm.

### 2.2. Butterfly Network Topology

Butterfly network [23–25] from the hypercube network is a hypercube deformation network.

**Figure 2:**
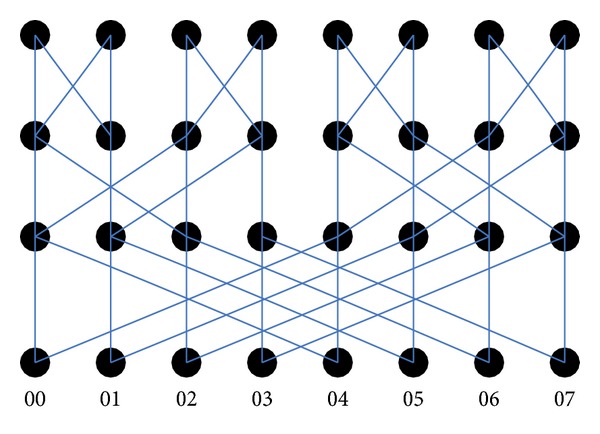
Three-layer butterfly network topology.

*n*-Dimensional butterfly network can be written as BF(*n*), and it exists as a vertex set, specifically indicated as shown in (1) in [26]:

(1) $$\begin{array}{c} V=\left\{\left(x;i\right):x\in V\left(Qn\right),\;\;0\leq i\leq n\right\}, \end{array}$$

where *n* is the number of layers with the butterfly network denoted by BF(*n*) of the butterfly network with *n* + 1 layers. *x* is the horizontal coordinate of the butterfly network. When the value of *n* is determined, the maximum value of *x* will be determined specifically for 2^*n*^. *Qn* is the set of nodes for each layer.  Figure 2 shows a three-layer butterfly network.

Known butterfly network layer parameters *n* can determine the width of the network that each node has the number of 2^*n*^, so a BF(*n*) network has (*n* + 1)2^*n*^ vertices. *n* is 3 in Figure 1, and the number of nodes in the network topology is 32.

If *j* = *i* + 1 and *x* = *y*, or *x* and *y* have exactly *j* different coordinates, (*x*, *i*) and (*y*, *j*) vertices are interconnected by an undirected edge.

When *x* = *y*, the side is called the direct side, and the remaining sides are called cross-edges.

In the butterfly network BF(*n*), each vertex of the 0-layer and *n*-layers is 2, and the remaining vertices are 4, so the entire network has the formula for calculating the number of edges as shown in

(2) $$\begin{array}{c} e\left(\text{BF}\left(n\right)\right)=2\times n\times 2^{n}=n\times 2^{n+1}. \end{array}$$

As the butterfly network and hypercube networks are very similar, so they have the following advantages. (1) Butterfly network has a simple recursive structure; that is, BF(*n*) network can be simply divided into two disjoint BF(*n* − 1) networks. Two disjoint BF(*n* − 1) networks will be able to get by removing all the vertices of the *n*th layer BF(*n*). (2) The unique path length *n* exists between (*x*, 0) and (*y*, *n*) vertices, it is just a vertex which passes through each layer, and the use of cross-edges from the *i* to *i* + 1 layer only has exactly *i* + 1 different coordinates. So we can know that BF(*n*) has *n*-order.

The butterfly network structure has good performance, so it can host multiple source nodes, and it can give the full advantages of a high number of routes, and reducing network latency, overhead, and so forth performs well. However, it does not have path diversity, in adversarial networks or high load on the network congestion situation which is more serious.

### 2.3. BFC Network Topology

The analysis from the previous section can know that butterfly network [27] can give the full advantages of a high number of routes, but it does not have path diversity in dealing with congestion in poor performance. Clos network has a good path diversity, which can provide multiple data link between each pair of nodes, so it has a good solution to the problem of network congestion. However, the routing process must use a lot of middle-class switching module, which needs more access lines resulting in much higher routing delay than the butterfly network, but the network overhead is larger than the butterfly network.

This section presents that the BFC topology network has a combination of the above two advantages, while overcoming their shortcomings. It is derived from the butterfly network inheriting the excellent network performance of the butterfly network; you can use a high number of routing equipment; the routing latency is low it also has the advantages of Clos network path diversity.

**Figure 3:**
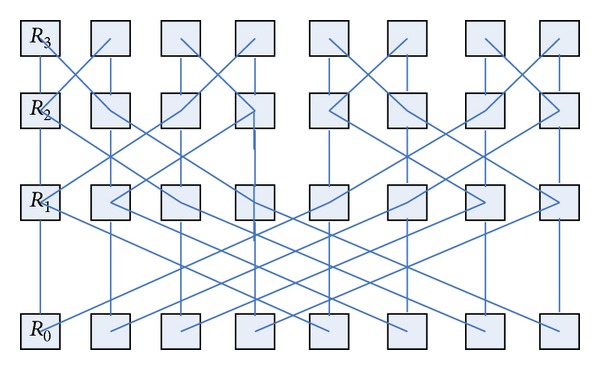
Three-node butterfly network routing plans.

**Figure 4:**
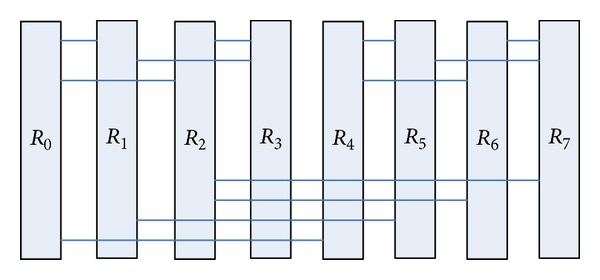
Planar butterfly network.

BFC network layer of the butterfly network with a number of different dimensions of the node modules is integrated into a new module. The exchange of information between different layers uses a two-way link to complete in the new unified network.  Figure 3 shows a three-layer butterfly network; each node is a routing node; they can connect a number of resource nodes. Four routing nodes *R*_0_, *R*_1_, *R*_2_, and *R*_3_ in the leftmost of Figure 3 are integrated into a single routing node, and the remaining nodes are integrated with the same approach, so we can get the routing node map shown in Figure 4. This transformation forming graph is also known as the planar butterfly network [28]. In the new network topology, the merger of the four routing information transmission between nodes is done directly in the internal nodes, data transfer between nodes using the combined data link transmission. The link is bidirectional, which can satisfy the input and output.

**Figure 5:**
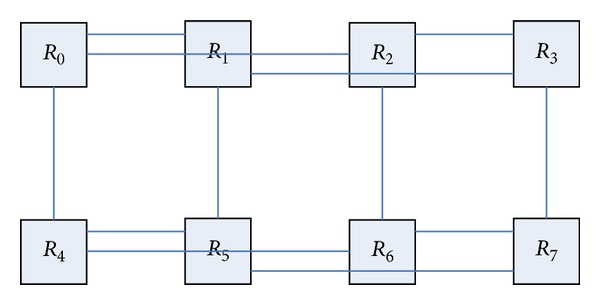
Planar butterfly network after changing the topology distribution.

The structure shown in Figure 4 for a certain topology planning can form a network structure shown in Figure 5.

**Figure 6:**
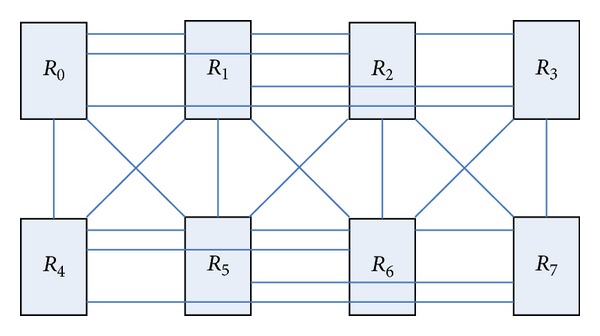
BFC network topology.

Figure 5 shows that the routing node *R*_0_, respectively, interconnects with routing nodes *R*_1_, *R*_2_, and *R*_4_. We make some improvements on the network structure, so that it has symmetry and better path diversity.  Figure 6 is the improved network topology-BFC network structure, so butterfly network through several transformations will gradually evolve into BFC network structure.

As can be seen from Figure 6 that the improved routing node *R*_0_ have existed the channels with *R*_1_, *R*_2_, *R*_3_, *R*_4_, and *R*_5_, which greatly increases the network path diversity, which can effectively reduce the network congestion. While the network also has a symmetry, which is more scalable.

### 2.4. BFC Topology Performance Analysis

This section is the performance analysis and comparison from the path diversity, scalability, network diameter, and energy consumption in terms of BFC topology and other common topologies.

#### 2.4.1. Path Diversity

**Figure 7:**
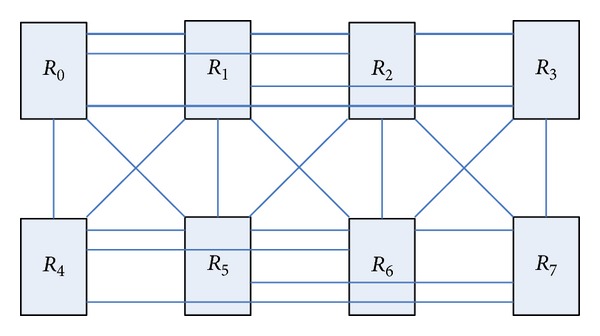
Path diversity of BFC network.

Since BFC network topology inherits the characteristics of Clos network, it also has a path diversity. As shown in Figure 7 when a data packet is transported from the routing node *R*_0_ to *R*_2_, the transmission path exists as follows:

(3) $$\begin{array}{c} R_{0}⟶R_{1}⟶R_{2} \end{array}$$

Or

(4) $$\begin{array}{c} R_{0}⟶R_{3}⟶R_{2} \end{array}$$

When the transfer process needs not to consider the shortest path, BFC provides network routing path that will be more and more even with the use of non-shortest-path routing algorithm, and it can be circuitous transmission by the nodes of the next level, or it can also be back and forth transmission between layers.

#### 2.4.2. Scalability

**Figure 8:**
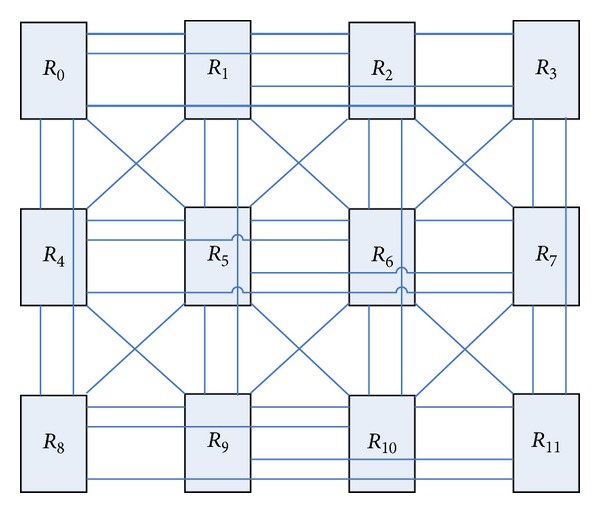
Expanded BFC network.

From the previous section we can see that butterfly network has a good scalability by the improved Planar butterfly network. Add a new route node, but it lies with its rows and columns of all the nodes connected, but also it will diagonally connect the nodes to complete the expansion of the network. Compared to the butterfly network, BFC network scalability is more favorable.  Figure 8 is the BFC network topology expanded from 8 nodes to 12 nodes.

**Figure 9:**
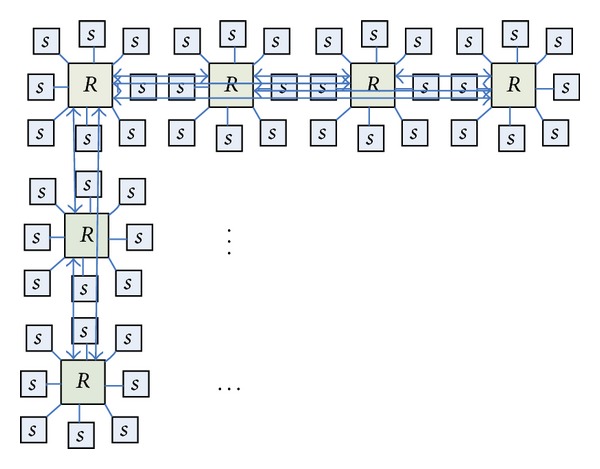
BFC network topology connected to 8 resource nodes.

**Figure 10:**
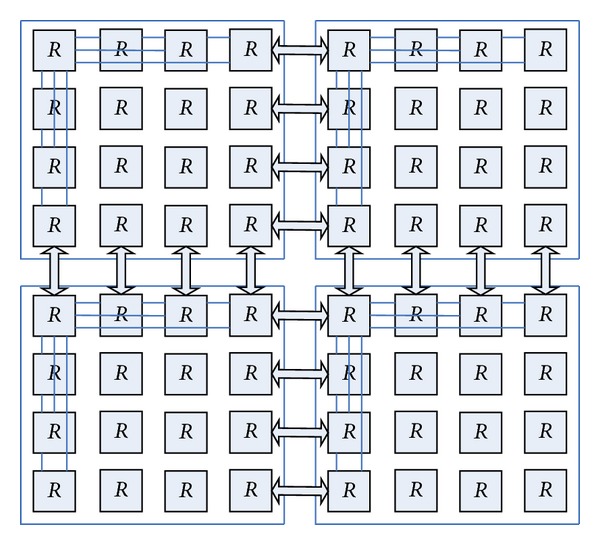
Expanded BFC network coupled with Mesh structure.

For BFC network that can accommodate more nodes, in addition to the above method for the horizontal expansion of the network, there are several other aspects that may be considered. (1) A single routing node is connected to increase the number of resource nodes, such that the original network routing nodes are connected from 4 resource nodes increased to 8 resource nodes shown in Figure 9. As the number of resource nodes increases, the corresponding link bandwidth also needs to make appropriate improvements to accommodate increased data traffic. (2) Several BFC networks can be coupled together according to certain structure. There are some limitations using the lateral extension method after the routing nodes increased, because the corresponding link bandwidth between two nodes becomes less and less; then the data transfer rate and the delay will increase. There can be a few same BFC-scale network coupled together using a network topology, such as using Mesh structure coupled together, so as to increase the cost of the average number of hops to reduce the adverse effects of narrow channels, and the 25 specific implementation is shown in Figures 9 and 10.

#### 2.4.3. Network Diameter

In the 4 × 4 network topology, the network diameter of Mesh network structure is 6; the network diameter of Torus structure is 3; the network diameter of butterfly network is 3; the network diameter of BFC network structure is 6. Therefore, the network diameter of BFC network topology is greater than the butterfly network.

#### 2.4.4. Transmission Delay

BFC network provides a number of direct lines, the BFC network connection can be routed directly, and the butterfly network may need to jump three times. And because BFC network has the path diversity, and the handling capacity of the network congestion is better than the butterfly network, so its transmission delay is less than the butterfly network. This end-to-end delay in the simulation can demonstrate the advantages of BFC in this regard.

#### 2.4.5. Network Throughput

For a network topology 4 × 4 = 16 nodes, it can be obtained according to formula (5). The throughput performance comparison of different topologies is shown in Table 1. The throughput of the Mesh structure and the butterfly network structure is minimum; Octagon and Torus network throughput is followed; the BFC network throughput is the largest. Because the BFC network inherits the path diversity of the Clos network; it has strong processing power for the congestion and very good throughput performance:

(5) $$\begin{array}{c} \text{TH}\leq \frac{2b\times \text{Bc}}{N}. \end{array}$$

#### 2.4.6. Chip Power Consumption

As NoC is based on nanoscale network and the network size is very large, so the network's energy consumption has become a major constraining factor in network performance. Because the power not only affects the life of the chip, but also closely relates to the amount of heat networks, such a small amount of heat corresponding to the chip must also be small; otherwise it is easy to accumulate chips burn calories.

Energy consumption of NoC generally consists of three parts. The data sheet needs the energy *P*_*L*_ from the source node to destination node in the routing chain process. The data sheet needs the energy *P*_*M*_ on the device forward in the buffer. The data sheet needs the energy *P*_*R*_ of data routing and switching module.

Specific formula is shown in the below equation:

(6) $$\begin{array}{c} \text{Power}=P_{L}+P_{M}+P_{R}. \end{array}$$

**Figure 11:**
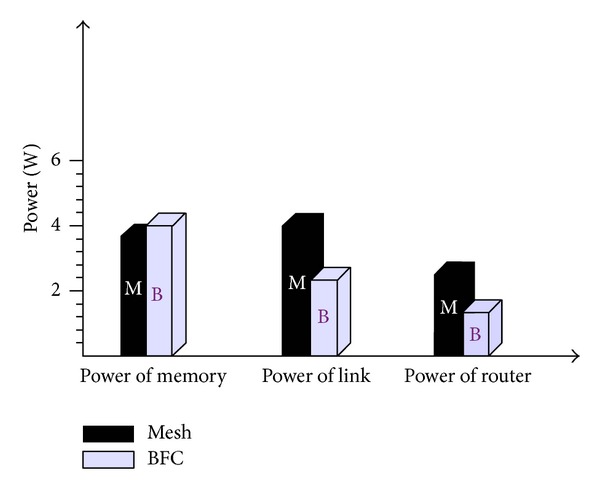
Comparison of two networks energy consumption.

As the BFC network provides the direct link between the same dimension nodes and the cross-links between the different dimension nodes. Compared with the Mesh topology, the required data transmission link length and the power consumption *P*_*L*_ is smaller. There is only one link between nodes in Mesh structure, but the bandwidth of the same dimension is used by many links simultaneously in the BFC structure, so as to reduce the bandwidth of a single link. Thus, in the routing process, crossbar switch module required for conversion also reduces the amount of data, and the routing process *P*_*R*_ energy consumption also is decreased. Since, in the BFC structure, each node corresponds to a number of links, so each node corresponds to the number of link input and output buffer more than the Mesh structure. So the energy *P*_*M*_ required buffer devices higher than Mesh, but due to reduction of bandwidth of each link, you can switch to smaller capacity cache device to indirectly reduce network energy consumption. The comparison of the specific energy consumption of the network is shown in Figure 11.

## 3. Routing Algorithm Design

This section designs a deterministic deadlock-free routing algorithm for BFC network by comparing the size of vertical and horizontal coordinates of the current node and destination node to determine the output port.

**Figure 12:**
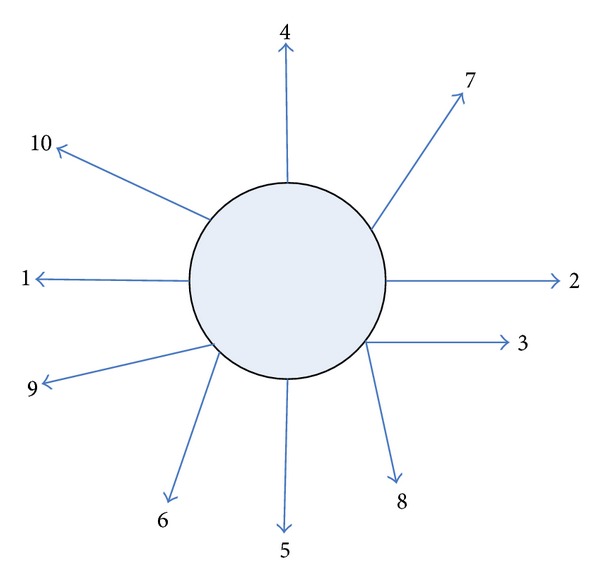
Routing port layout.

In the topology of *N* = 16 routing number, the network will be placed in the coordinate system, and each router has a corresponding coordinate values (*x*, *y*). There are four diagonal nodes and four vertical and horizontal nodes, and the requirements connected with the IP core router port number are 0. The nodes of *X*-axis direction are corresponding to the ports 1, 2, and 3. The port number is 4, 5, 6 in the *Y*-axis direction. Four port numbers of the right diagonal clockwise rotation nodes are 7, 8, 9, and 10 as shown in Figure 12.

Set the current node coordinates *C*(*cx*, *cy*), the target node coordinates *D*(*dx*, *dy*), and the output port outport.

Routing algorithm is described as pseudocode.

 When the routing receives a packet, examine the packet header containing the destination node to compute the coordinate difference between the target node and the current node: *X* = *dx* − *cx*, *Y* = *dy* − *cy*.
 When *X* == 0 and *Y* == 0, it indicates that the data packets reach the destination node, outport = 0.
 When *Y* == 0 and *X* > 0, it indicates the packet's destination node in the right direction of the current node, and we choose the right direction of the port output.
 When *Y* == 0 and *X* < 0, it indicates the packet's destination node in the left direction of the current node, and we choose the left direction of the port output.
 When *Y* < 0 and *X* == 0, it indicates the packet's destination node under the direction of the current node, and we choose the next direction of the port output.
 When *Y* < 0 and *X* > 0, it indicates the packet's destination node in the direction of the current node, the choice of the direction of the port output.
 When *X* > 0 and *Y* > 0, it indicates the packet's destination node at the top right of the current node to select the top right to the port output.
 When *Y* > 0 and *X* < 0, it indicates the packet's destination node at the top left of the current node to select the upper left to the port output.
 When *Y* < 0 and *X* > 0, it indicates the packet's destination node in the lower right of the current node to select the port for output to the lower right.
 When *X* < 0 and *Y* < 0, it indicates the packet's destination node in the left bottom of the current node to select the lower left to the port output.

Algorithm pseudocode is shown in Algorithm 1.

The algorithm limits the direction of the routing data packets in the current node and the destination node forms a square area routes, and the direction must always be toward the destination node; then this would limit the generation of the ring; thereby it damages the necessary condition for the formation of a deadlock. Thus, the routing algorithm is deadlock-free.

## 4. Simulation and Analysis

### 4.1. Simulation Setup

This paper uses simulation software OPNET simulating the performance of the BFC network, which can make us understand the pros and cons of their performance through comparison with the butterfly network.

OPNET has some important features, and a brief introduction is here. OPNET simulation of the entire design process is carried out around the object. Simulation model is based on the object as a unit to build up. OPNET has its own unique C-like language, and its syntax is similar to C language. It also has its own unique core function library, and the library functions begin with “op_”, which is similar to API functions in VC, and it is mainly used in the process model and the transceiver pipeline stage calls. It is divided by function into several different set of functions, and the same function names in a function have the same prefix. In using OPNET simulation process, we can use it for program debugging tool that comes with the test, and you can also call VC for joint commissioning, it is very convenient to people who are familiar with VC debugging environment.

These two concepts of the simulation time and the elapsed time need distinguished in the use.

Simulation time is the event of the system running in a simulated environment. Simulation's role in the operation of the core is to maintain the event list, and the table is based on the program listing. Each table represents an event, and that is an interrupt. Each event has its own serial number and the corresponding execution time. When it's on an event handler, it may remove it from the list and begin processing the next interrupt after the emulation core. If the simulation time of the events is 00:01, and the simulation time of this incident is 00:05, then the simulation time goes from 01 time to 05 times, but in reality, the process may only take 1 second.

Elapsed time is the actual physical time that the simulation takes in the real world. Executing the event does not require any time, and the simulation time may not be spent between events, but the physical time does not be consumed. Event execution until the event has finished executing, and the simulation time may not consume, but the actual physical time has been consumed.

In the simulation, we compare the performance of the three-tier butterfly network topology and the similar number of nodes in the node 36 BFC network routing. The analyzed network performance parameters are end-to-end delay and throughput. Since the variable rate is still selected into the network. In the simulation, the use of three traffic patterns, namely, uniform flow, hot flow, and matrix flow.

### 4.2. Simulation Results and Analysis

**Figure 13:**
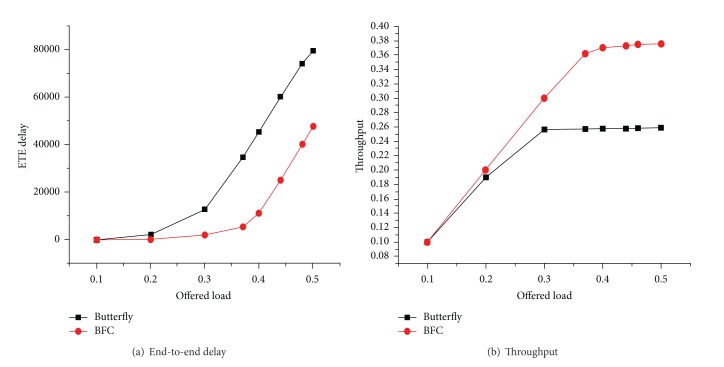
Performance comparison of two uniform traffic models.

Figure 13 shows the performance comparison in the uniform flow network corresponding to end-to-end delay and throughput of the butterfly network and BFC network. It can be seen that performance of the BFC network is better than the butterfly network. For end-to-end delay, as shown in Figure 13(a), the butterfly network in the injection rate reaches 0.2 when it has begun to rise, while the BFC network began to rise in the injection rate sustaining to 0.35. The number of BFC network links is more, and the advantage of rich path diversity shows up here. Throughput performance is similar to end-to-end delay as shown in Figure 13(b), and the throughput and saturation corresponding injection rate of BFC network are much higher than the butterfly network, and the reach saturation is also higher than the butterfly network.

**Figure 14:**
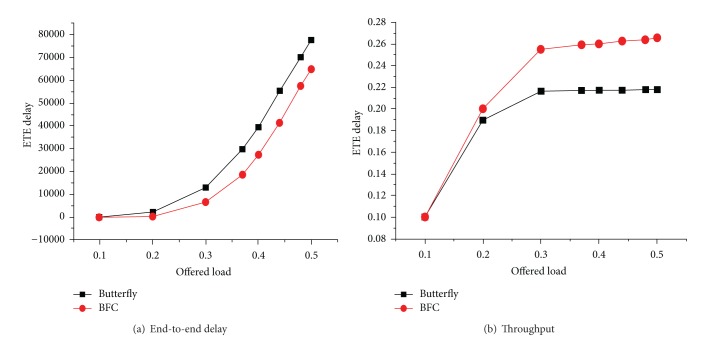
Performance comparison of two matrix flow models.

Figure 14 shows the performance comparison in the matrix flow network corresponding to end-to-end delay and throughput of the butterfly network and BFC network. The performance BFC network is still slightly better than the butterfly network, but the performance gap is not large. Two networks in the injection rate reach 0.2; and the latency begins to increase; the increase in the butterfly network trend is more obvious as shown in Figure 14(a). Throughput and latency have similar trends, and BFC network reaching the saturation point is higher than the butterfly network as shown in Figure 14(b).

**Figure 15:**
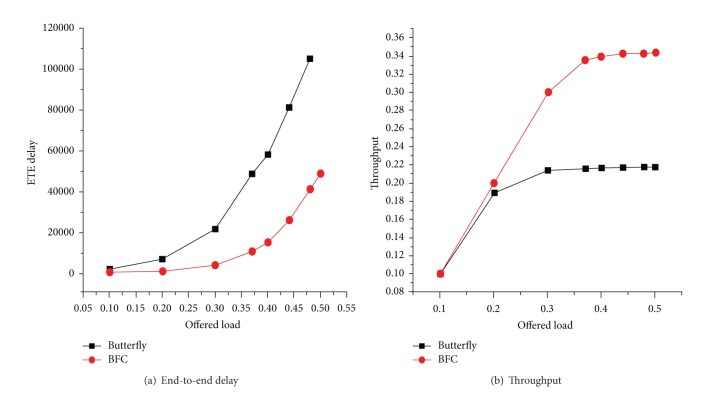
Performance comparison of two hot spots traffic models.

Figure 15 shows the performance comparison in the hot spots flow network corresponding to end-to-end delay and throughput of the butterfly network and BFC network. Hot spots traffic patterns are the common processing mode to investigate the ability of commonly used network. In this mode, the weakness of low processing capabilities with congestion of the butterfly network is exposed. For end-to-end delay as shown in Figure 15(a), the butterfly network almost reaches 0.17 from the injection rate when it begins to increase, while BFC starts up from the injection rate around 0.3. Throughput performance and end-to-end delay are similar as shown in Figure 15(b), and the butterfly network saturation throughput is much lower than the BFC network.

## 5. Conclusion

This paper in-depth studies the butterfly network and its advantages and disadvantages, and the new network topology, BFC, network is proposed with the combination of the characteristics of the Clos network structure and the butterfly network structure. The network originates in the butterfly network, and it can give full play to the advantages of a high number of routes with the characteristics of the low latency and overhead of the butterfly network, which overcomes the low processing power shortcomings of the butterfly network congestion due to the introduction of the structural characteristics of the Clos network, and it has a better path diversity. In addition, the network absorbs the characteristics of the Clos network, and it provides path diversity, and because it is based on the butterfly network, there is no middle class compared with the Clos network, so it is superior to Clos network in terms of latency or in the overhead area network equipment. The structure of the BFC network has a reasonable change, and the corresponding routing algorithm is proposed for its structural characteristics. Simulation results show that the topology has better latency and throughput than the butterfly network.

## Figures and Tables

**Algorithm 1:**
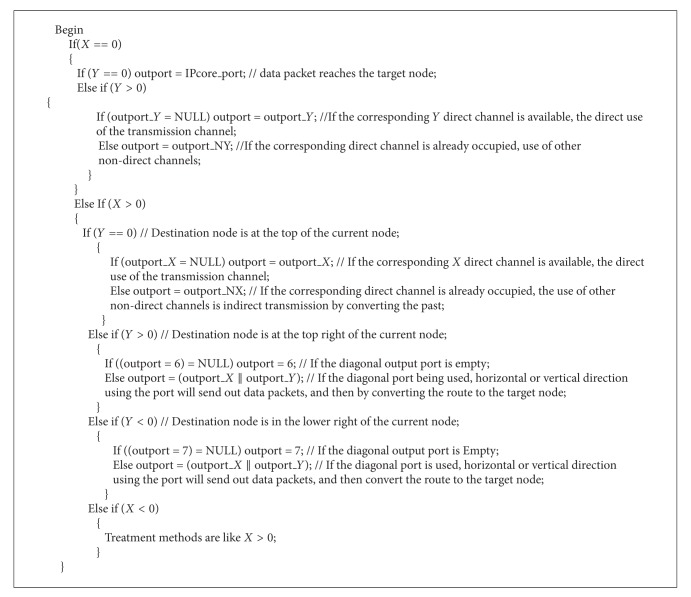


**Table 1:** Comparison of ideal throughput.

Topology	Bc	Throughput value
Mesh	8	TH_Mesh_ ≤ *b*
Torus	16	TH_Torus_ ≤ 2*b*
Octagon	12	TH_Octagon_ ≤ 3*b*/2
Butterfly	8	TH_Butterfly_ ≤ *b*
BFC	32	TH_BFC_ ≤ 4*b*
